# Medical News and Items

**Published:** 1876-01

**Authors:** 


### Medical News and Items.

INTERNATIONAL MEDICAL CONGRESS.—The Centennial Medical Commission is organized with the following officers:

President — Samuel D. Gross, M.D., LL.D., D.C.L. Oxon.

Vice-Presidents — W. S. W. Ruschenberger, M.D., U. S. N.; Alfred Stillé, M.D.

Recording Secretary—William B. Atkinson, M.D.

American Corresponding Secretaries—Daniel G. Brinton, M.D.; William Goodell, M.D.

Foreign Corresponding Secretaries—Richard J. Dunglison, M.D.; R. M. Bertolet, M.D.

Treasurer—Caspar Wister, M.D.

Arrangements have been made for the holding of the Congress in the city of Philadelphia. The Commission propose the following general plan for the organization and business of the Congress:

I. The Congress shall consist of delegates, American and foreign, the former representing the American Medical Association and the State and Territorial Medical Societies of the Union; the latter the principal medical societies of other countries.

II. The officers shall consist of a President, ten Vice-Presidents, four Secretaries, a Treasurer, and a Committee of publication, to be elected by the Congress at its first session, on the report of a Committee of Nomination.

III. The morning sessions of the Congress shall be devoted to general business and the reading of discourses; the afternoons to the meetings of the Sections, of which there shall be nine, viz: 1. Medicine, including Pathology, Pathological Anatomy and Therapeutics; 2. Biology, including Anatomy, Histology, Physiology and Microscopy; 3. Surgery; 4. Dermatology and Syphilology; 5. Obstetrics and Diseases of Women and Children; 6. Chemistry, Toxicology and Medical Jurisprudence; 7. Sanitary Science, including Hygiene and Medical Statistics; 8. Ophthalmology and Otology; 9. Mental Diseases.

Gentlemen intending to made communications upon scientific subjects will please notify the Commission at the earliest practicable date, in order that places may be assigned them on the programme.

The Congress will be formally opened at noon, on Monday, the fourth day of September, 1876, and close Sept. 9th. The registration book will be open daily from Thursday, Aug. 31, from 12 to 3 P. M., in the Hall of the College of Physicians, northeast corner 13th and Locust streets. Credentials must in every case be presented.

All communications must be addressed to the appropriate Secretaries: William B. Atkinson, 1400 Pine street, Philadelphia, Recording Secretary; Daniel G. Brinton, 2027 Arch street, William Goodell, 20th and Hamilton streets, American Corresponding Secretaries.

---

We are pleased to announce that our quarterly cotemporary, heretofore published in this city, changes its name with the new year, and will hereafter be known as the *Journal of Nervous and Mental Disease.* It will be issued simultaneously in Chicago and New York. Messrs. G. P. Putnam's Sons, of the last named city, taking charge of its issue there. Its editorial management will remain unchanged, as all readers of the former journal will be pleased to hear, but the co-operative assistance has been secured of Dr. Wm. A. Hammond, of New York; Dr. S. Weir Mitchell, of Philadelphia; and Dr. E. H. Clarke, of Boston.. The journal is to be also increased in size. It has already become the exponent of the American school of neurologists, and has secured a recognition abroad in which we are proud to participate.

